# Computational Investigations of the Water Structure at the α-Al_2_O_3_(0001)–Water Interface

**DOI:** 10.1021/acs.jpcc.3c03243

**Published:** 2023-07-27

**Authors:** Xiaoliu Zhang, Christopher G. Arges, Revati Kumar

**Affiliations:** † Department of Chemistry, Louisiana State University, Baton Rouge, Louisiana 70803-1804, United States; ‡ Department of Chemical Engineering, Pennsylvania State University, University Park, Pennsylvania 16802, United States

## Abstract

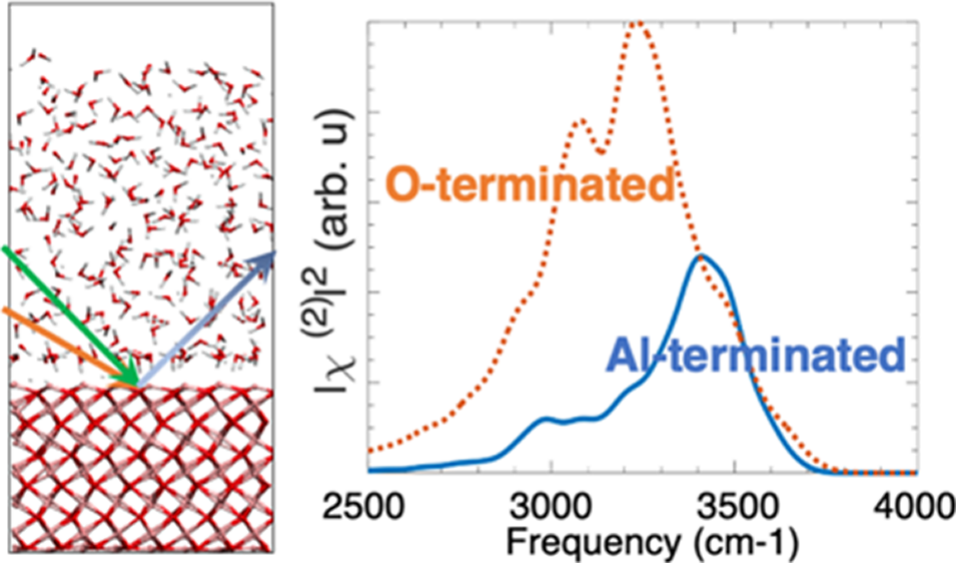

The α-Al_2_O_3_(0001)–water interface is investigated using *ab initio* molecular dynamics (AIMD) simulations. The spectral signatures of the vibrational sum frequency generation (vSFG) spectra of OH stretching mode for water molecules at the interface are related to the interfacial water orientation, hydrogen bond network, and water dissociation process at different water/alumina interfaces. Significant differences are found between alumina surfaces at different hydroxylation levels, namely, Al-terminated and O-terminated α-Al_2_O_3_(0001). By calculating the vibrational sum frequency generation spectrum and its imaginary component from AIMD results, the structure of interfacial waters as well as the termination of alumina slab are related to the spectral signatures of vSFG data.

## 1 Introduction

Oxide–water surfaces are relevant to a broad range of physicochemical phenomena and have applications in climate science, ocean chemistry, catalysis, electrochemistry, and gas sensing.^1−4^ In particular, the alumina–water interface is extensively investigated not only as a model system for the organization of water at oxide–water interfaces but also due to the wide application of alumina surfaces in various fields from environmental science to microelectronics.^1,5−9^ Among the different phases of aluminum oxide, the α-Al_2_O_3_(0001) surface has been the focus of a number of studies since it is the most stable of the alumina surfaces. In addition, the high density of aluminol groups on this surface provides an opportunity to look into the interaction between the surface aluminol groups and interfacial water molecules.^10^ The water dissociation process at this interface has been widely investigated experimentally and computationally, including for use in bipolar membranes used in pH adjusting process streams with electrodialysis^11^ and membrane capacitive deionization^12^ and electrolyzers that convert water into green hydrogen^13^ and carbon dioxide into value-added products.^14^ Previous studies of water dissociation at surface aluminol groups have often resulted in controversies. For instance, free energy profiles along proton transfer pathways on the α-Al_2_O_3_(0001)–water interface determined from molecular simulations have shown that the dissociated states of water are preferred over molecularly adsorbed at the alumina–water interface.^15^ The spectra from some vibrational sum frequency generation (SFG) experiments appear to confirm this, as inferred from the significant blue-shift of the OH stretch signal, which is believed to arise from the surface aluminol (Al-OH) groups resulting from water dissociation.^16−19^ On the other hand, in another set of SFG studies as well as the infrared reflection absorption spectroscopy studies, these blue-shifted OH signals are not observed.^20−22^ Recent work by Yue et al. pointed out that the experimental investigations employed different sample preparation techniques, which in turn can modify the surface structure of the alumina slab.^23^ In addition, they revealed that once the alumina surface is created, the Al-terminated surface and the Gibbsite-like surface do not interconvert.^23^ In order to help solve these seemingly opposing observations, high-level ab initio molecular dynamics of the α-Al_2_O_3_(0001)–water system for two limiting surface terminations have been carried out here to characterize the surface structure of these systems. These results will be especially relevant to the use of alumina in next general bipolar membranes as water dissociation catalysts to aid in pH modulation for selective ionic separations.

*Ab initio* molecular dynamics (AIMD) simulations can, in principle, enable one to obtain a complete picture of the interfacial structure and dynamics as well as the reactivity at the molecular level. By calculating vSFG spectra from the AIMD results, one can bridge the gap between the molecular structure as well as the chemical phenomena that take place at the alumina–water interfaces and the spectral signatures in the vSFG lineshape. To this end, two different α-Al_2_O_3_(0001) surfaces, namely, Al-terminated α-Al_2_O_3_(0001) surface and O-terminated α-Al_2_O_3_(0001) surface, are simulated and compared. The Al-terminated α-Al_2_O_3_(0001) surface is fully dehydroxylated, while the O-terminated α-Al_2_O_3_(0001) surface is fully hydroxylated.

The study is divided into the following sections. Section 2 outlines the computational methodology including the ab initio molecular dynamics (MD) simulation setup . The results are discussed in Section 3, and the conclusions are presented in Section 4.

## 2 Computational Details

The two kinds of α-Al_2_O_3_(0001) interfaces described above were modeled using systems comprising a six-layer alumina slab (13 Å) with a layer of water molecules (20 Å) and vacuum (70 Å) for a total thickness of 100 Å. The (0001) Al-terminated surface was cleaved from the unit cell of the ideal *R*3*c* crystal structure taken from Materials Project.^24−38^ The ideal model of the fully hydroxylated (O-terminated) surface was created by replacing each outermost Al atom with three H atoms^39^(see Supporting Information Figure S1). The hexagonal unit cells of two surfaces were then expanded along *x* and *y* axes to construct a 4 × 4 supercell, 19 Å on each side. The alumina–water interface was generated by adding 233 water molecules on top of the alumina slab. All simulations were performed under periodic boundary conditions (PBC). The construction of the slab, as well as the alumina–water interface, was performed using the GROMACS software.^40^

Classical MD simulations of both interfaces were carried out prior to AIMD to generate initial configurations using the GROMACS software^40^ with a classical force field, namely, CLAYFF^41^ for alumina and SPC/E for water molecules.^42^ The cutoff radius for short-range electrostatic interactions and van der Waals interactions was set to 7.5 Å. The electrostatic interaction was simulated using the Ewald method,^43^ while the van der Waals interaction was computed using 12-6 Lennard-Jones (LJ) potential with the Lorentz–Berthelot combining rule.^44^ For both sets of simulations, the system first underwent energy minimization, followed by equilibration in the *NPT* ensemble (2 ns with a timestep of 0.4 fs, pressure of 1.0 bar using the Berendsen barostat, and a temperature of 300 K using a Nosé–Hoover thermostat) followed by production runs in the *NVT* ensemble (20 ns production run with a timestep of 0.4 fs and a temperature of 300 K) with a Nosé–Hoover thermostat.^45−47^

A series of AIMD simulations for the two systems (5 simulations for each case) were performed at room temperature using the CP2K package^48^ at the density functional theory (DFT) level of theory. The revPBE functional was used along with the empirical D3 dispersion correction, and the DZVP-MOLOPT-SR basis set with GTH pseudopotentials.^49−54^ The revPBE functional was chosen because it reproduces the vSFG spectrum of water at both the air–water as well as the graphene oxide–water interface.^55^ Initial configurations for AIMD simulations were obtained by taking five random snapshots separated by more than 200 ps from the production run of a classical MD simulation. Hence, for each system, five different AIMD simulations were carried out (starting from the dynamically uncorrelated configurations from the classical MD simulation) in order to ensure adequate sampling. For each system, and for each initial configuration generated from the classical MD simulation, energy optimization and cell relaxation were performed using the L-BFGS algorithm,^56^ followed by a 5 ps equilibration run and a 20 ps production run using the canonical (*NVT*) ensemble at 300 K with a timestep of 0.5 fs.

## 3 Results and Discussion

### 3.1 Average Water Density Fluctuations from the Instantaneous Water Interface

**Figure 1:**
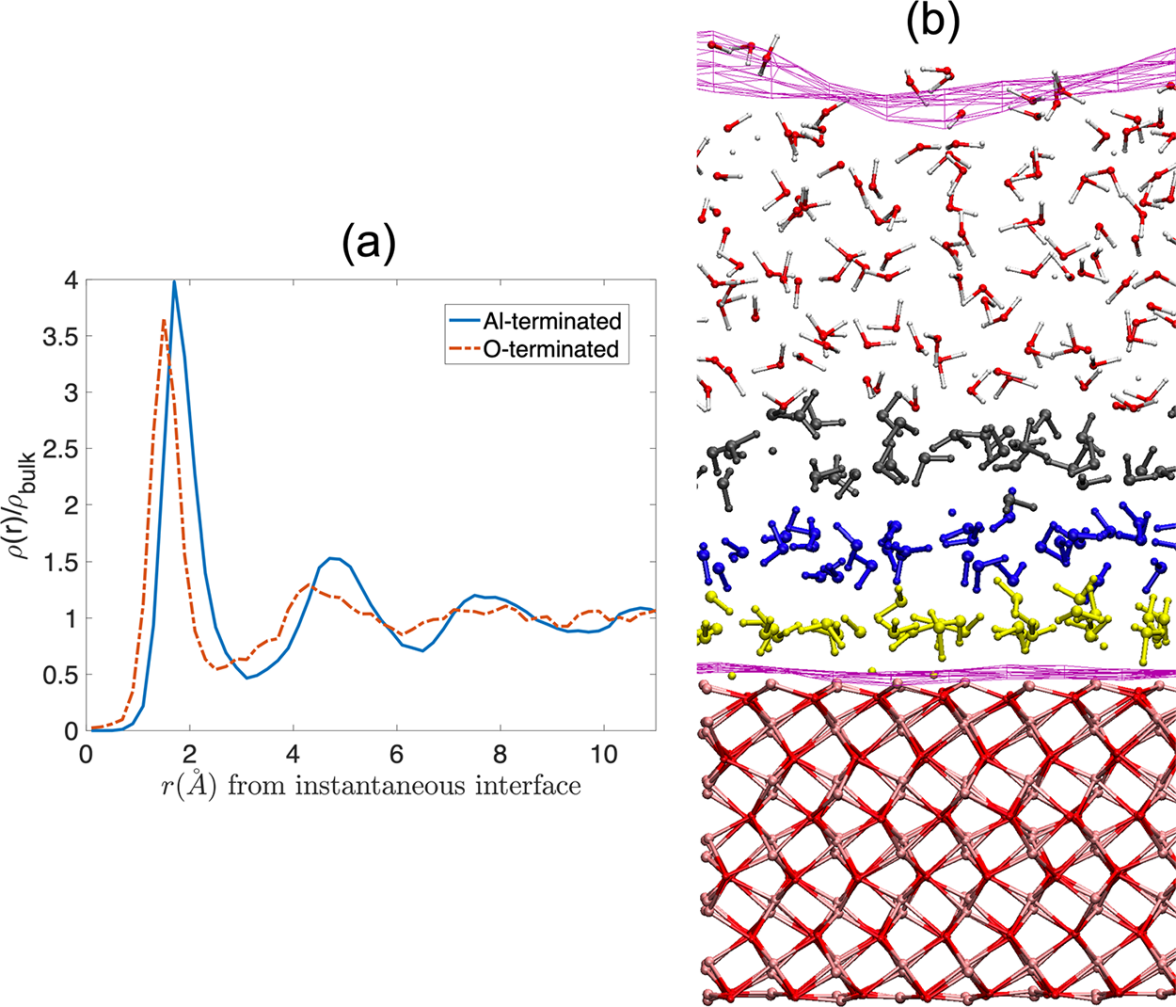
(a) Ratio of the density of water to the bulk density of water as a function of the distance to the instantaneous water interface at the alumina surface (note: the ratio goes to 1 when one reaches bulk water); (b) Representation of the Al-terminated Al_2_O_3_/H_2_O system with the instantaneous water surface (purple grid), Al_2_O_3_ slab (O atoms in red and Al atoms in pink), and three different water layers (yellow: L1; blue: L2; gray: L3) as well as the water-air interface (and its associated instantaneous surface).

The interfacial water structure was first investigated by calculating the water density profile at the alumina/water interface. The ratio of the water density to the bulk water density was calculated as a function of the distance to the Willard–Chandler instantaneous water interface^57,58^ and is shown in Figure 1a. Three distinct water layers, namely, L1, L2, and L3, corresponding to the three local minima in the density can be observed for both systems, with the L1 as the first dominant interfacial layer. A representation of the Al-terminated Al_2_O_3_/H_2_O system with the instantaneous water surface (purple grid), Al_2_O_3_ slab (O atoms in red and Al atoms in pink), and three different water layers is shown in Figure 1b.

In the Al-terminated system (Figure 1a, blue solid line), the first and second local minimum are located at 3.1 and 6.5 *Å* respectively, while in the O-terminated system (Figure 1a, red dash line), the first and second local minimum are located at 2.5 and 6.1 *Å*. The first maxima in the Al-terminated and O-terminated systems are at 1.7 and 1.5 *Å*, respectively. Therefore, the L1 interfacial layer in the Al-terminated system is thicker and further away from the instantaneous interface compared with the O-terminated system, while the L2 layer is relatively thicker in the O-terminated system. These differences in the L1 region arise because the L1 in Al-terminated system is a complex mixture of water molecules adsorbed on surface O atoms (see Supporting Information Figure S3a), water molecules adsorbed on the surface Al atoms (Figure S3b), adsorbate hydroxide ions (Figure S3c), water adsorbed on surface aluminol (Figure S3d), and water molecules forming hydrogen bonds with the adsorbed water (Figure S3e). On the other hand, the L1 in the O-terminated system is only composed of water molecules that form hydrogen bonds with either the O atoms or the H atoms of the surface OH (Figure S3f and g). The former (see Figure S3f) is found to be the dominant species and the latter (Figure S3g) is rarely seen in the L1 layer, which agrees with the water orientation results (in section c) and will be discussed further in section c. In addition, the water density profile of the Al-terminated system has more pronounced peaks for the L2 and L3 water layers, indicating more structuring in the L2 and L3 water layers in this case as compared to the O-terminated system.

### 3.2 Simulated vSFG Spectra of the Alumina–Water Interface

The vibrational SFG (vSFG) spectra of the alumina–water interface were calculated via the surface specific velocity–velocity correlation function (ssVVCF) formalism.^59^ The resonant component of the second-order response function χ^(2)^ is as follows:
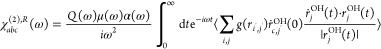
1here, *r*_*j*_^OH^ is the *j*th water OH vector and *ṙ*_*j*_^OH^ is the corresponding velocity. *Q*(ω) is the quantum correction factor and is given by
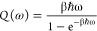
2where  and *ℏ* is the reduced Planck’s constant. A smoothing Hann window function is applied to the Fourier transform with the cutoff parameter set as 0.5 ps.

The intra-/intermolecular coupling is considered by introducing the cross-correlation terms controlled by the switching function:
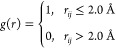
3where *r_ij_* is the distance between the center of mass of the O–H stretch chromophores *i* and *j*.

The non-Condon effects are included by replacing the frequency-independent transition dipole moment (transition polarizability) with the frequency-dependent transition dipole moment μ(ω) (frequency-dependent transition polarizability α(ω)) parameterized by Corcelli and Skinner:^60,61^
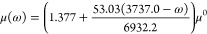
4
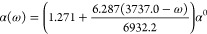
5

**Figure 2:**
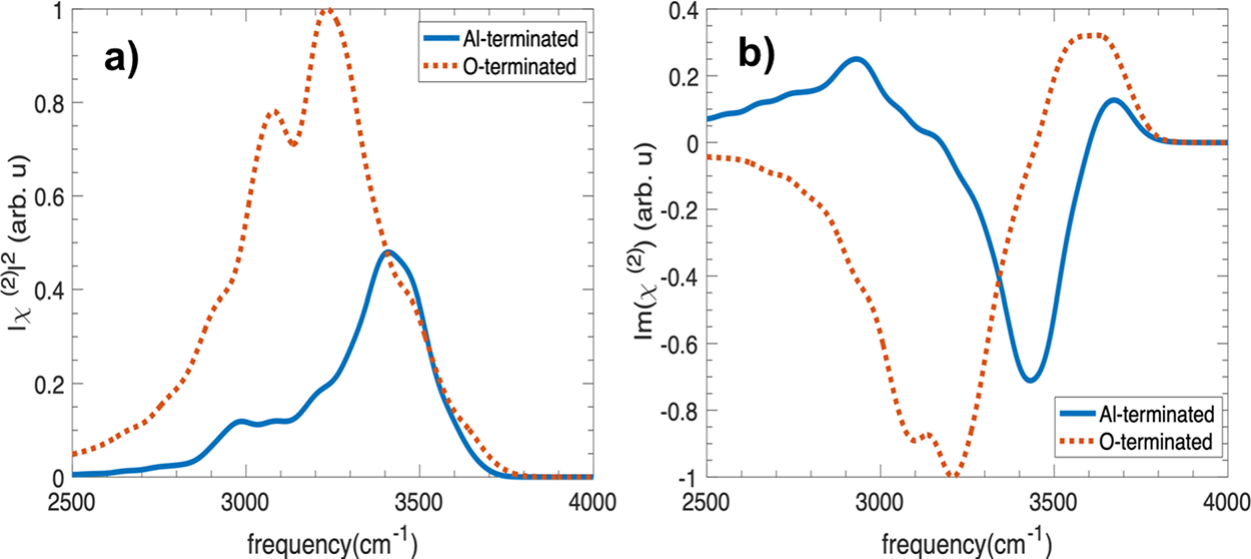
(a) Simulated vSFG spectra of the OH stretching mode of water molecules in the L1 layer and (b) the corresponding imaginary part for the Al-terminated (solid blue) and the O-terminated (dotted red) alumina–water interfaces.

VSFG spectra of the OH stretching mode was calculated for both systems from the interfacial water molecules in the L1 water layer (Figure 2). VSFG spectra calculated from water layer L2 (see Figure S2) and L3 are not discussed in this paper due to their extremely small intensities and thus negligible contributions to the total alumina–water vSFG spectra compared with the spectra from waters in the L1 layer. Arbitrary units were used to normalize the second-order response. In the case of the O-terminated system, there are two main peaks centered at 3100 and 3250 cm^–1^ respectively, with two shoulders at around 3450 and 2950 cm^–1^. In the Al-terminated system, there is one large central peak centered at 3450 cm^–1^ and a much smaller broad peak centered at around 3000 cm^–1^. The 3200 cm^–1^ peak, the 3450 cm^–1^ peak, and the 3000 cm^–1^ broad peak were observed in previous experimental vSFG results,^62,63^ which indicates that the interface prepared in the experiment is a mixture of Al-terminated and O-terminated alumina–water interface. Based on the literature, the peaks at 3200 and 3450 cm^–1^ are determined to be related to different hydrogen bonding environments around the water molecules.^20,64,65^ DelloStritto et al. assigned the peak centered at 3450 cm^–1^ from their AIMD simulations to the surface OH groups and the peak at 3150 cm^–1^ to the contribution from the surface water molecules.^62^ However, in the work by Gaigeot et al., both water and “in-plane” aluminols contribute to the 3400 cm^–1^ peak, while “out-of-plane” aluminol vibrations are at a higher frequency, between 3600 and 3800 cm^–1^.^5^ Here, the “in-plane” and “out-of-plane” aluminols are the surface aluminols parallel and perpendicular to the water–alumina interface, respectively.

In this work, the vSFG spectra is obtained using the OH stretch modes that belong to water molecules at the beginning of the simulation, namely, at the start of the production run (after 5 ps of equilibration in the *NVT* ensemble that follows cell optimization). The spectra were first compared to what has been previously published in the literature.^21,62,63^ First, as mentioned previously DelloStritto et al. assigned the peak at 3450 cm^–1^ to surface O–H groups (their AIMD simulations were solely on O-terminated slabs),^62^ which would indicate that for the Al-terminated system (which has the main peak at 3450 cm^–1^), almost all the water molecules in the water layer L1 should be dissociated on the alumina/water interface once alumina is exposed to liquid water. However, this is not seen in the AIMD simulations in this study. Second, for the Al-terminated alumina/water system, smaller and less well-defined peaks are observed in the frequency range from 2700 to 3000 cm^–1^, which can be related to the delocalized proton^66^ resulting from the water dissociation process at the interface. Finally, in previous vSFG experiments, the surface hydroxylation is not specially controlled in the sample preparation process nor is it measured. Considering that the ratio of the intensities of the peaks centered at 3150 and 3450 cm^–1^ in the experimental results (∼0.8) and the ratio of these two peaks in simulated results (∼2.2), one can infer that less than 30% of the alumina surface undergoing the sample preparation process described in previous literature is hydroxylated with ∼70% of the surface sites staying dehydroxylated.^62^ This result is in agreement with the obvious broad peak centered at 3000 cm^–1^ in the experimental spectra and the previous studies on alumina terminal interconversion.^23,62^

To further investigate the spectral signatures of the vSFG spectra and connect it to the first layer water structure, the resonant imaginary component, Imχ_*xxz*_^(2)^, was examined for the two systems at different surface hydroxylation levels. The sign of the imaginary part reflects the direction of the transition dipole of the O–H stretch with respect to the interface, where a positive sign corresponds to the OH bond with the H atom pointing away from the interface, and a negative sign corresponds to an OH bond with H pointing toward the interface.^67^Figure 2b shows the imaginary component Imχ_*xxz*_^(2)^ for the Al-terminated and O-terminated alumina/water interfaces. In the frequency region between 3450 and 3800 cm^–1^, the O-terminated system shows a broad positive peak centered at 3600 cm^–1^, while the Al-terminated system shows a sharper negative peak centered at 3450 cm^–1^ and another weaker positive peak at 3650 cm^–1^, indicating a larger distribution of interfacial water orientations in the Al-terminated system when compared to the O-terminated system. In the frequency region between 2500 and 3450 cm^–1^, the O-terminated system shows a large negative peak centered at 3200 cm^–1^ with a shoulder at 3100 cm^–1^, while the Al-terminated system shows a broader positive peak at 3000 cm^–1^.

### 3.3 Interfacial Water Orientation and Hydrogen Bond Analysis

**Figure 3:**
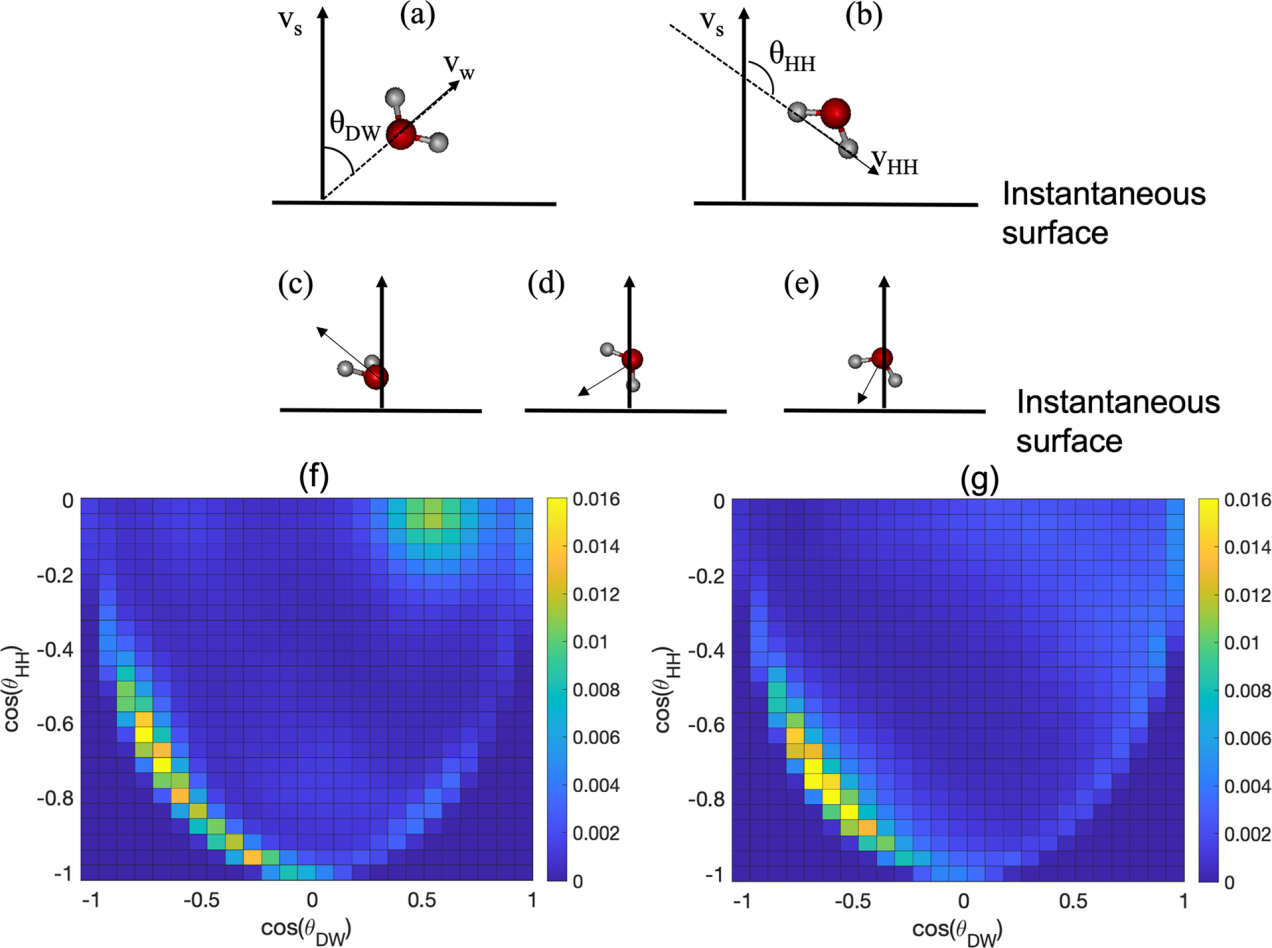
(a) Definition of the water orientation angle (θ_DW_). *V*_W_ is the water dipole vector and *V*_S_ is the normal vector of the instantaneous surface. (b) Definition of the water orientation angle (θ_HH_). *V*_HH_ is the vector connecting the two hydrogen atoms of a water molecule vector (pointing from the hydrogen which is further away from the surface to the hydrogen atom close to the surface). (c) Representation of the water molecule with two OH oscillators pointing up (2-up). (d) representation of the water molecule with one OH pointing up and one OH pointing down (1-up,1-down). (e) Representation of the water molecule with both the OH oscillators pointing down (2-down). (f) Two-dimensional histograms of the joint distribution of the cos(θ_DW_) and the cos(θ_HH_) of water molecules in the L1 system for the Al-terminated Al_2_O_3_/H_2_O system; (g) two-dimensional histograms of the joint distribution of the cos (θ_DW_) and the cos(θ_HH_) of water molecules in the L1 system for the O-terminated Al_2_O_3_/H_2_O system.

To interpret the vSFG spectral signatures and to capture the different interfacial water structures in the two systems, the distribution of the orientation of water molecules in the L1 layer was calculated. Here, two angular order parameters, namely, θ_DW_, defined as the angle between the dipole vector of the water molecule and the normal vector of the interface pointing toward the water layer (see Figure 3a), and θ_HH_, defined as the angle between the vector connecting the two hydrogen atoms of the water molecule (pointing toward the interface) and the normal vector of the surface in the direction of the water layer (see Figure 3b), were examined. The joint distribution of cos(θ_DW_)/cos(θ_HH_) for water molecules in the L1 region for the Al-terminated system and O-terminated system are shown in Figure 3f,g, respectively. Both systems show a distribution centered when both angles are at around 140°. This broad distribution corresponds to the water molecules with one OH pointing toward the instantaneous surface and the other OH either pointing toward (2-down) or away from the instantaneous surface (1-up, 1-down) (see Figure 3d,e). However, the Al-terminated system also prefers a water orientation with θ_HH_ at 90° and θ_DW_ at around 50°. The corresponding structure is shown in Figure 3c, where both OHs in the water molecule are pointing away from the interface (2-up). This finding agrees with the positive peak in vSFG resonant imaginary component Imχ_*xxz*_^(2)^ in the Al-terminated system at the frequency range between 2500 and 3300 cm^–1^, while the O-terminated interface shows a negative peak at the same frequency range.

While the positive resonant imaginary component in the Al-terminated system at low frequency clearly arises from the interfacial waters with both of their OH pointing away from the interface, the complexity of Imχ_*xxz*_^(2)^ in this system at the high frequency region requires further discussion. Therefore, the imaginary component Imχ_*xxz*_^(2)^ arising from interfacial water molecules with different orientations was examined along with their hydrogen bonding environments.

**Figure 4:**
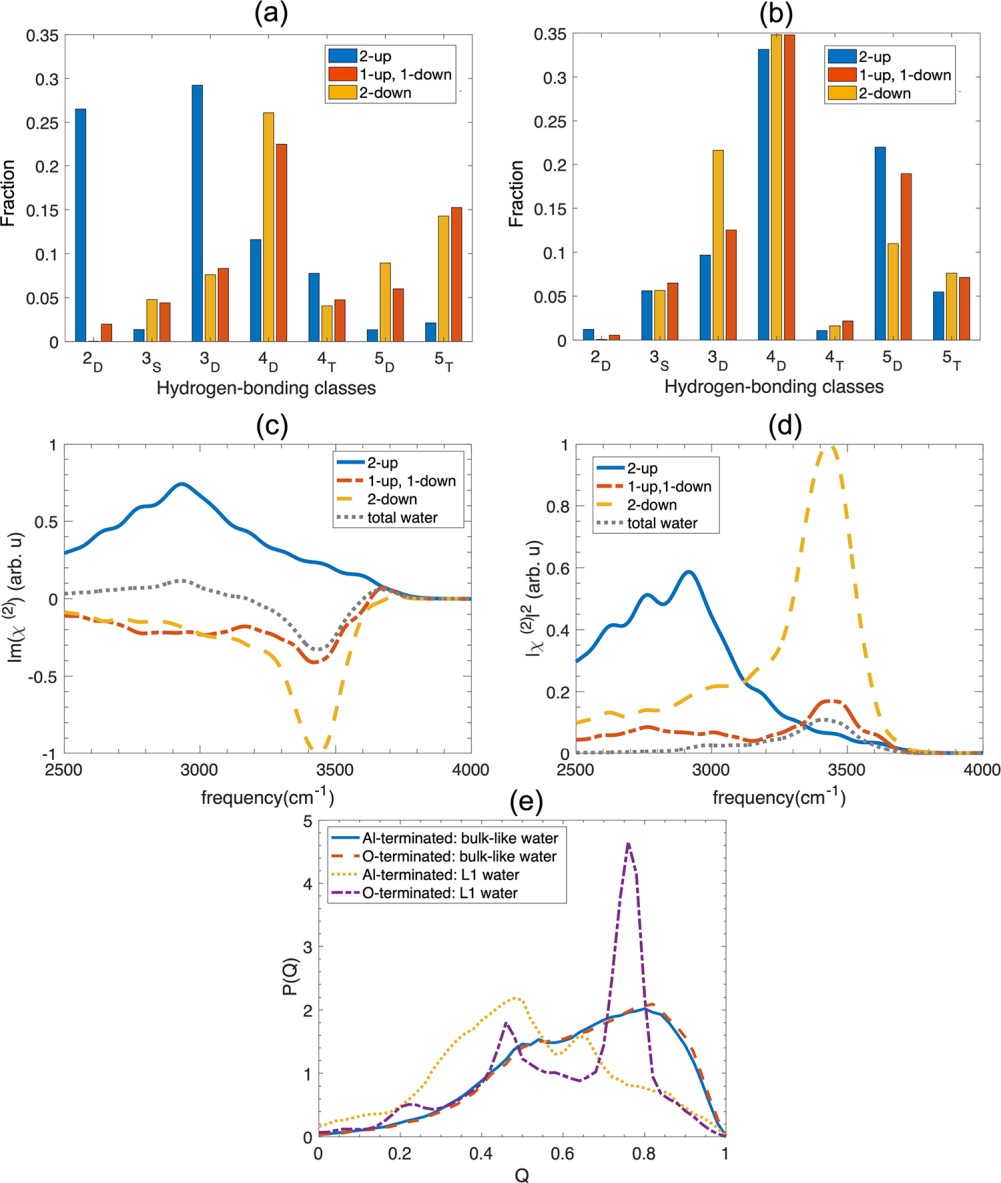
Distribution of the different hydrogen bonding classes of water molecules in the L1 layer for (a) Al-terminated and (b) O-terminated systems for water orientations 2-up (blue), 1-up, 1-down (red) and 2-down (yellow). (c) Imaginary part of the simulated vSFG spectra of OH stretching for 2-up (blue), (1-up, 1-down) (red), and 2-down (yellow) water in layer L1 at the Al-terminated alumina–water interface. (d) Simulated vSFG spectra for 2-up (blue), 1-up, 1-down (red), and 2-down (yellow) water in layer L1 at the Al-terminated alumina–water interface. (e) Distribution of the tetrahedral order parameter of waters in the L1 region as well as the bulklike region for the Al- and O-terminated systems.

The hydrogen bonding environment of water molecules is evaluated using a naming scheme adopted from the work by Auer et al.,^68^ wherein water is defined as residing in a hydrogen-bonding class *N*_a_, where *N* represents the total number of hydrogen bonds a water molecule is involved in, and the subscript letter (a = S/D/T/Q) refers to the number of hydrogen bonds for which water under consideration acts as a hydrogen bond donor. Here, S is for a single donor water, D for a double donor water, and T and Q are for triple and quadruple donor waters, respectively. Water molecules are defined to form a hydrogen bond when the distance between the oxygen atom of the proton acceptor and the hydrogen atom of the proton donor is less than 2.5 *Å*.^69^Figure 4a,b shows the fraction of hydrogen-bonding classes for 2-up (blue), (1-up, 1-down) (red), and 2-down (yellow) water molecules within the L1 layer for Al- and O-terminated systems, respectively, and Figure 4c,d shows the imaginary part of the vSFG spectra due to these different waters for the two surfaces.

For the Al-terminated system, the environment of water molecules with two OH oscillators pointing away from the interface (2-up water) shows significant differences from those with one or two OH oscillators pointing toward the interface ((1-up, 1-down) water and 2-down water). The 2-up water has similar contributions from 2_D_ and 3_D_ types of water hydrogen bonding environments, while the major classes in the second and third case ((1-up, 1-down) and 2-down) are 4_D_, 3_D_, and 5_T_. For water molecules with both OH oscillators pointing up (2-up), the O atoms are either binding to surface Al atoms (e.g., 2_D_) or forming a hydrogen bond with the hydrogens in surface aluminols (e.g., 3_D_ and 4_T_), which gives rise to the relatively small number of hydrogen bonds that the O atoms of these 2-up water form with other waters. Meanwhile, water molecules with one OH pointing up and the other OH pointing down (1-up, 1-down) are mainly binding to surface O on alumina slab via hydrogen bonding. Hence, these oxygen atoms in the water molecules are accessible to nearby water molecules in the L1 layer and can act as a single or double acceptor, resulting in the major classes 3_D_, 4_D_, and 5_T_. For water molecules pointing down to the interface (2-down), 3_D_, 4_D_, and 5_T_ are the most common, and moreover, the proportion of 4_D_ and 5_D_ is larger compared to water molecules with one OH pointing up. Water molecules with this orientation (2-down) are closer to water layer L2, with their hydrogen atoms exposed to the other two kinds of waters in L1, while their oxygen atoms are accessible to water molecules in L2. Since the water molecules in L2 behave more like bulk water and are less organized as compared with L1, oxygen in those water molecules that point down mainly act as a double acceptor from nearby waters. This leads to a hydrogen bonding network that is closer to bulk water. For waters in the L1 layer of the O-terminated surface (see Figure 4b), the dominant hydrogen bonding environment for all the three types of waters is 4_D_. Figure 4e shows the distribution of the tetrahedral order parameter, *Q* (see Supporting Information Figure S4 for the definition),^70^ for the waters in the L1 layer and the bulklike layers for the Al- and O-terminated systems. The distribution for the O-terminated system has a sharp peak at 0.8, indicating a larger proportion of tetrahedral water (perfect tetrahedron corresponds to a value of 1), suggesting a more icelike structure, in keeping with 4_D_ being the dominant hydrogen bonding environment, whereas the peak is broader and shifted to lower values for the Al case.

Imχ_*xxz*_^(2)^ is decomposed by recalculating the Imχ_*xxz*_^(2)^ for each individual interfacial water molecule types in layer L1 of the Al-terminated system and is plotted in Figure 4. This includes the contribution from 2-up, (1-up, 1-down), and 2-down water molecules within the L1 layer for the Al-terminated system, respectively. The 2-down water shows a well-defined negative peak centered at 3450 cm^–1^, while a broad positive band centered at around 3000 cm^–1^ is observed for the 2-up water. This agrees with the statement that the sign of the imaginary component Imχ_*xxz*_^(2)^ is dependent on the orientation of the corresponding OH oscillator: OH pointing away from the surface (pointing up) results in a positive Imχ_*xxz*_^(2)^, whereas OH pointing toward the surface (pointing down) leads to a negative sign of Imχ_*xxz*_^(2)^. The (1-up, 1-down) water shows a major negative peak at the same frequency as 2-down water (but less intense) and two low positive peaks at the frequency of 3000 and 3700 cm^–1^. The reason could be that the positive signal generated from the OH oscillator pointing up cancels out with the negative signal from the OH pointing down at 3450 cm^–1^.

**Figure 5:**
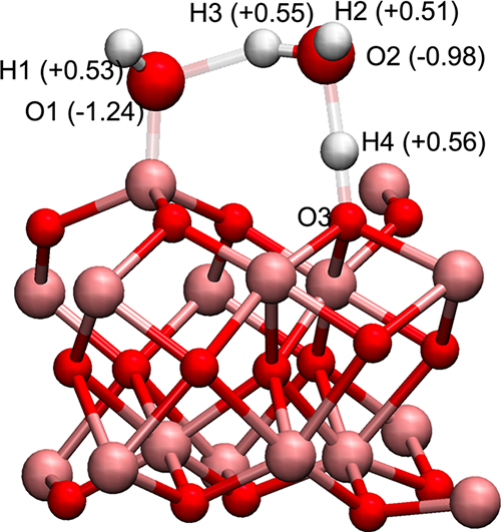
Snapshot of the alumina–water cluster taken from AIMD trajectory for DFT frequency calculation and NBO analysis. The calculated atomic charges are also shown on the relevant atoms.

Electronic structure calculations at the density functional (DFT) level (using the B3LYP functional^71−73^ and 6-31+g(d,p) basis set) have been carried out on a representative water–Al_2_O_3_ cluster to determine the O–H frequencies (see Table 1) so as provide further insight into the peaks for 2-up and (1-up, 1-down) waters. Figure 5 shows a water–Al_2_O_3_ cluster taken from AIMD trajectory, which results from the water dissociation process on the alumina–water interface and is only observed in the Al-terminated system. The atoms O1, H1, and H3 (see in Figure 5) originate from a 2-up water molecule, while O2, H4, and H2 constitute a (1-up, 1-down) water molecule. According to the results of the frequency calculation, on the optimized cluster (see Table 1 for results), the high frequency peak at 3700 cm^–1^ in 2-up water is assigned to the stretching mode of surface aluminols AlOH. The blue-shifted positive peak with small intensity at around 3700 cm^–1^ in (1-up, 1-down) water is assigned to the OH pointing up in the water molecule. These kinds of OH are also referred as “topmost dangling OH” in some previous studies.^18^ The 3700 cm^–1^ positive peaks are not obvious in the imaginary part of the vSFG spectra for the Al-terminated interface due to their relative low intensity (Table 1) caused by the small transition dipole and the small concentration due to the formation of the hydrogen bond with other surrounding water molecules to form 2_D_ and 4_D_ molecules. However, the peak is confirmed to be real by calculating the vSFG spectra of the surface aluminol groups in the slab for the O-terminated system (Figure S5), where only a small proportion of its surface aluminol groups form hydrogen bonds with water molecules. The vSFG spectra of the surface aluminol groups in the O-terminated system show a few shoulder peaks at around 3400 to 3600 cm^–1^, which can arise from the hydrogen bonding between the surface aluminol and the water molecules. This result agrees with previous studies that the surface aluminol Al_2_(OH) also contributes to the 3400 cm^–1^ peak in the O-terminated system.^63^ However, this structure does not contribute much to the 3400 cm^–1^ peak in the Al-terminated system according to Figure 4c,d. Moreover, since the structure of the Al-terminated slab has only one layer of Al at the surface (Al-O1-O2-Al1-Al2-Al-), the surface aluminols are coordinated with only one Al (namely, AlOH), resulting in a perpendicular orientation (“out of plane”) relative to the interface, which agrees with the work of Gaigeot et al. that “out-of-plane” aluminol vibrations are between 3600 and 3800 cm^–1^.^5^ Charges from natural bond orbital (NBO) analysis on the alumina–water cluster taken from AIMD trajectory (shown in Figure 5) indicate that the hydroxylate group in surface aluminol AlOH is negatively charged (Figure 5). This charge analysis suggests the existence of the interfacial hydronium H3O+ (O2, H2, H3, and H4) and polarizable hydroxide ion OH– (O1H1), resulting from the water dissociation process. The Grotthuss proton (H3) in the hydronium is delocalized between the hydronium O (O2) and the hydroxide O (O1) and thus gives rise to the positive peaks centered at around 3000 cm^–1^.^74^ Hence, the broad linewidth of the 3000 cm^–1^ peak in the AIMD spectrum confirms the proton delocalization, which is essential for the interfacial water dissociation process and surface proton transfer. Representative clusters for the undissociated 2_D_ and 3_D_ waters that involve delocalized protons were also chosen from the trajectory, and the frequencies were determined (see the Supporting Information for details, Figures S6, S7, and Table S1). The calculated frequencies for those waters with their full solvation shell confirmed the assignment of the broad peak at 3000 cm^–1^. The negative peak at around 3450 cm^–1^ is assigned to the more bulklike, 4_D_, 5_T_, and 5_D_ water molecules, which are not hydrogen-bonded with atoms in the slab. It should be noted that the structure of water molecules at the Al-terminated interface is less tetrahedral when compared with the water molecules at the O-terminated interface (dominantly “icelike” 4_D_ water), and hence, the latter generates a vSFG signal centered at around 3150 cm^–1^. These assignments of peaks are in agreement with the work of Zhang and co-workers.^18^ Moreover, the minor intensity at the low frequency (2500 cm^–1^) is related to the delocalized protons in the surface aluminols (Al_3_OH) formed by surface oxygen atoms and dissociated water protons.

**Table 1:** Results of DFT Frequency Calculation on the Alumina–Water Cluster Taken from AIMD Trajectory

frequency (cm^–1^)	intensity	OH stretch mode
2506	1910	O3H4
2926	570	O2H3
3887	76	O2H2
3890	66	O1H1

**Figure 6:**
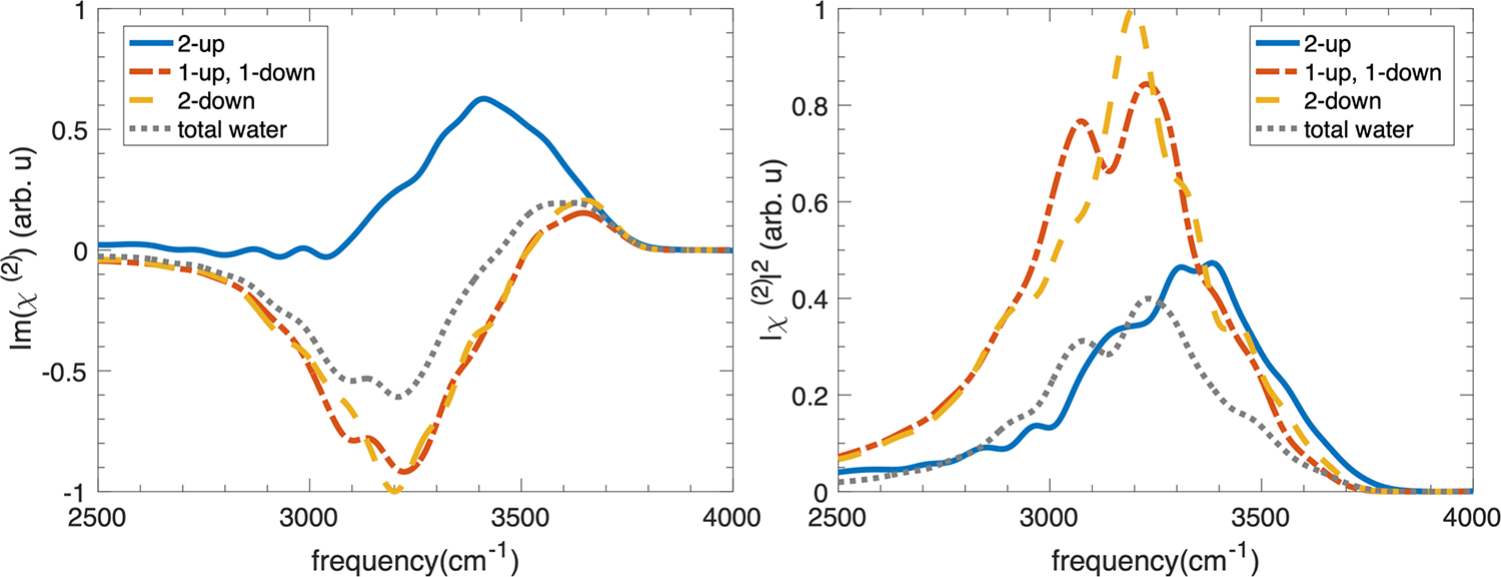
Simulated vSFG spectra (right) and their imaginary part (left) of OH stretching mode in 2-up (blue), (1-up, 1-down) (red) and 2-down (yellow) water in layer L1 at the O-terminated alumina–water interface.

Hydrogen bonding classes and decomposition of SFG spectra have been calculated for L1 waters in the O-terminated system for comparison (Figures 4b and 6). Unlike the Al-terminated system, where water molecules with different orientations show significant differences in hydrogen-bonding classes, here, the 4_D_ structure is the dominant environment, irrespective of the orientation. This indicates a more tetrahedral and icelike structure in the L1 layer for the O-terminated system. From Figure 3g, it is clear that waters with both OH pointing down (cos(θ_DW_) < −0.62) or both OH pointing up (cos(θ_DW_) > +0.62) are rare. As for the vSFG spectra, the signals related to the delocalized proton in water dissociation events (around 6 events are seen on average for each of the five AIMD simulations), which gives rise to the broad positive peak centered at around 3000 cm^–1^ in the Al-terminated case, is not observed in the imaginary part of SFG spectra calculated from the O-terminated system. 2-down and (1-up, 1-down) water structures generate SFG signals centered at around 3150 cm^–1^ and are close to the SFG signal of liquid-like water with a small red-shift, in keeping with the distribution of hydrogen bonding classes (4_D_ structure is dominant). Furthermore, these are in agreement with peak assignments in previous studies that related the signals at 3140 and 3450 cm^–1^ to the tetrahedrally (icelike) and nontetrahedrally bonded water molecules, respectively.^18,20^ However, although the molecular environment of the three water structures are similar based on the hydrogen-bonding class analyses, there are small frequency shifts (around 150 cm^–1^) among the three classes of water structures. These frequency shifts may arise from different hydrogen bonding strengths. Strong hydrogen bonding with alumina oxygen will cause red-shift in the water OH stretch compared to the bulk. This 3450 cm^–1^ positive peak is not obvious in the spectra of total water because the 2-up waters at the O-terminated alumina–water interface are relatively rare, as can be inferred from Figure 3f,g.

## 4 Conclusions

The SFG spectra were obtained from AIMD simulations with the revPBE-D3 functional using the method of Ohto et al.^59^ Different water layer structures at the alumina–water interface in Al-terminated and O-terminated systems were compared. The main signatures of the SFG spectra were connected to the solvation environment of the interfacial water molecules, namely, orientation, hydrogen-bonding classes, and water dissociation (see a summary in Table 2). Positive peaks centered at 3700 cm^–1^ are assigned to “out of plane” surface aluminols related to the water dissociation process, while the broad positive peaks from 2500 to 3500 cm^–1^ are assigned to adsorbate water molecules (1-up, 1-down) and 2-up. Peaks centered at 3150 and 3450 cm^–1^ are water molecules with different hydrogen bonding environments. The former has more icelike tetrahedral structure, while the latter exhibits a more nontetrahedral solvation environment. These results provide significant insight for the characterization of the hydroxylation level of alumina/water interface in experiments. Furthermore, the interfacial solvation environment and reactivity can inform the development of alumina-based water dissociation catalysts in bipolar membranes. In future work, the effect of applied electric fields to the water dissociation process will be examined, which should further help obtain molecular insight for the above application.

**Table 2:** Assignment of SFG Features and to OH Types and Their Orientation and H-Bonding Class

frequency (cm^–1^) centered at	OH type	orientation	HB class
Al-terminated			
∼3700	dangling surface aluminols (AlOH) formed from 2-up, and dangling OH in (1-up, 1-down) perpendicular to surface	2-up, (1-up, 1-down): OH up	2_D_ in 2-up
			3_D_, 4_D_ in (1-up, 1-down)
∼3450	OH in water molecules, hydrogen-bonded with other water molecules	(1-up, 1-down), 2-down	3_D_, 4_D_, 5_T_
∼3000 (broad peak)	delocalized protons	2-up	2_D_, 3_D_
∼2500 (broad peak)	delocalized protons	(1-up, 1-down): OH down	3_D_, 4_D_
O-terminated			
∼3150	OH in water molecules, hydrogen-bonded	(1-up, 1-down), 2-down	3_D_, 4_D_, 5_D_

## Supporting Information Available

Additional figures showing the different alumina terminations; water species in L1 in the two systems; definition of orientational tetrahedral order parameter *Q*; additional calculations on the frequency of the OH stretching mode under different solvation environments including simulated vSFG spectra of the surface aluminols at the O-terminated alumina–water interface; simulated vSFG spectra calculated from water layer L2; and the frequency calculations of the 2_D_ and 3_D_ water at the Al-terminated alumina–water interface (PDF)
