# Supplementary material for: Computational Investigations of the Water Structure at the α-Al2O3(0001)–Water Interface
Source: J Phys Chem C Nanomater Interfaces. 2023 Jul 27;127(31):15600–10. doi: 10.1021/acs.jpcc.3c03243 (PMC10428097; doi:10.1021/acs.jpcc.3c03243)
Supplement: Supplementary file 1 — jp3c03243_si_001.pdf [file jp3c03243_si_001.pdf]

Supporting Information

Computational Investigations of the Water Structure at the α-Al_2O_3 (0001)-Water Interface

Xiaoliu Zhang,[a] Christopher G. Arges,[b] and Revati Kumar[a]*

[a]*Department of Chemistry, Louisiana State University, Baton Rouge, Louisiana 70803-1804, United States*

[b]*Department of Chemical Engineering, Pennsylvania State University, University Park, Pennsylvania 16802, United States*

Table of content

1. α-Al2O3(0001) Slabs··························S3

2. vSFG spectra Calculated From Water Layer L2··························S4

3. Water Structures In Water Layer L1 At Alumina-Water Interfaces··········S5

4. Definition of Tetrahedral Order Parameter··························S6

5. Simulated vSFG spectra of the surface aluminols at the O-terminated alumina-water interface··························S7

6. Frequency calculations of the 2_D and 3_D water at the Al-terminated alumina-water interface··························S8

7. References ··························S10

1. α-Al2O3(0001) Slabs

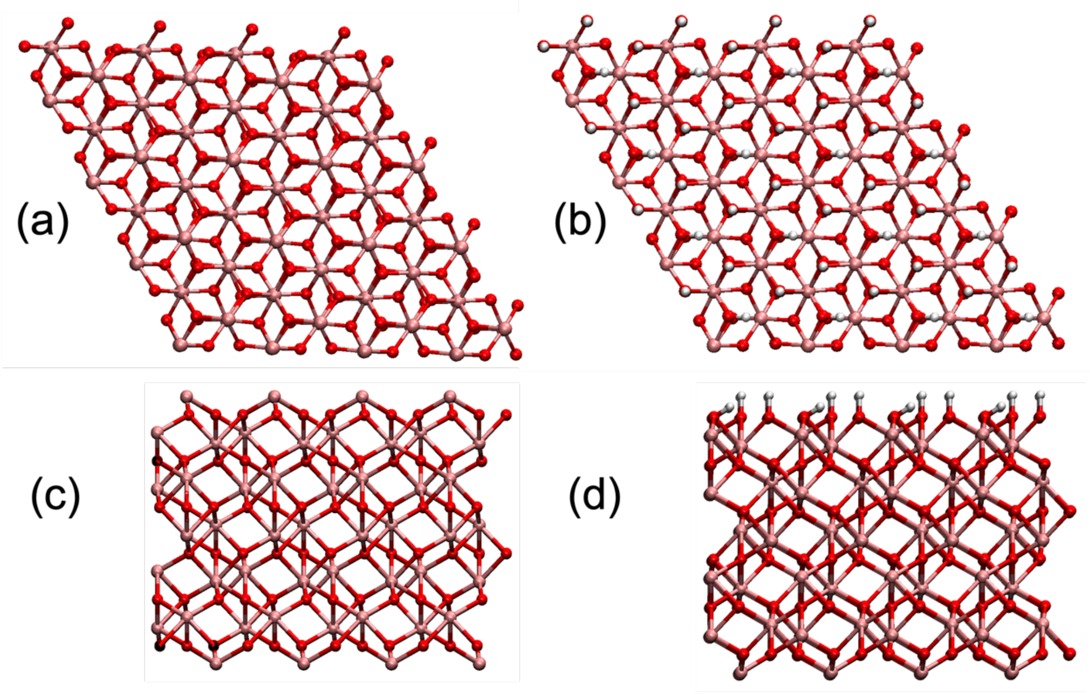

Figure S1. The top (top) and side view (bottom) of Al-terminated (left) and O-terminated (right) α-Al2O3(0001) slab. Al, O and H atoms are shown in pink, white and red, respectively

Al-terminated and O-terminated α-Al2O3(0001) slabs are shown in Figure S1. The fully hydroxylated (O-terminated) surface was created by replacing each outermost Al atom with three H atoms based on previous studies.[1]

2. vSFG spectra calculated from water layer L2

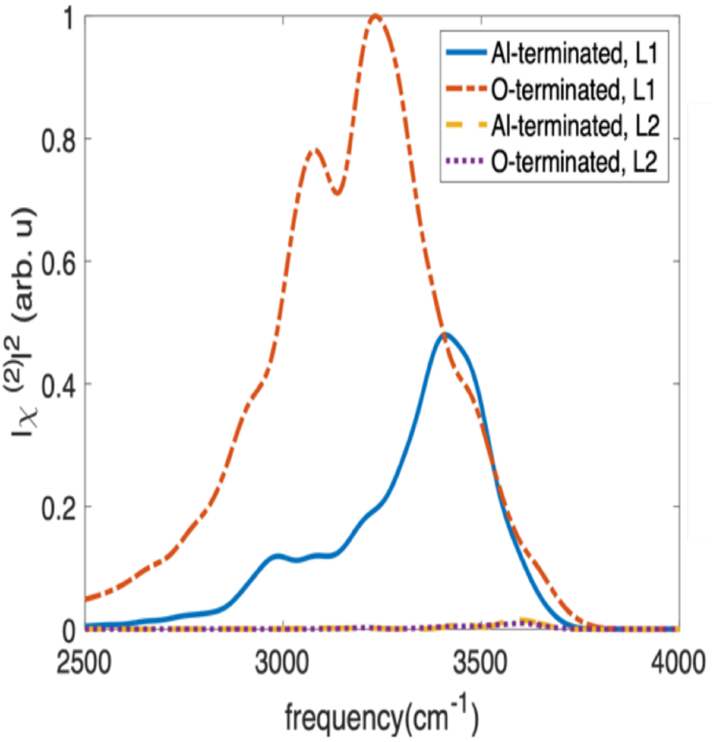

Figure S2. The vSFG spectra of water OH calculated from L1 and L2 in the Al-terminated (blue line) and O-terminated (red line) systems after normalization with respect to the spectra calculated from L1 water.

The comparison between vSFG spectra of water in L1 and L2 in both Al-terminated system and the O-terminated systems are shown in Figure S2. The spectra of L2 water is about 100 time less intense when comparing with that of L1 water, which results in negligible contribution to the total SFG spectra.

3. Water Structures In Water Layer L1 At Alumina-Water Interfaces

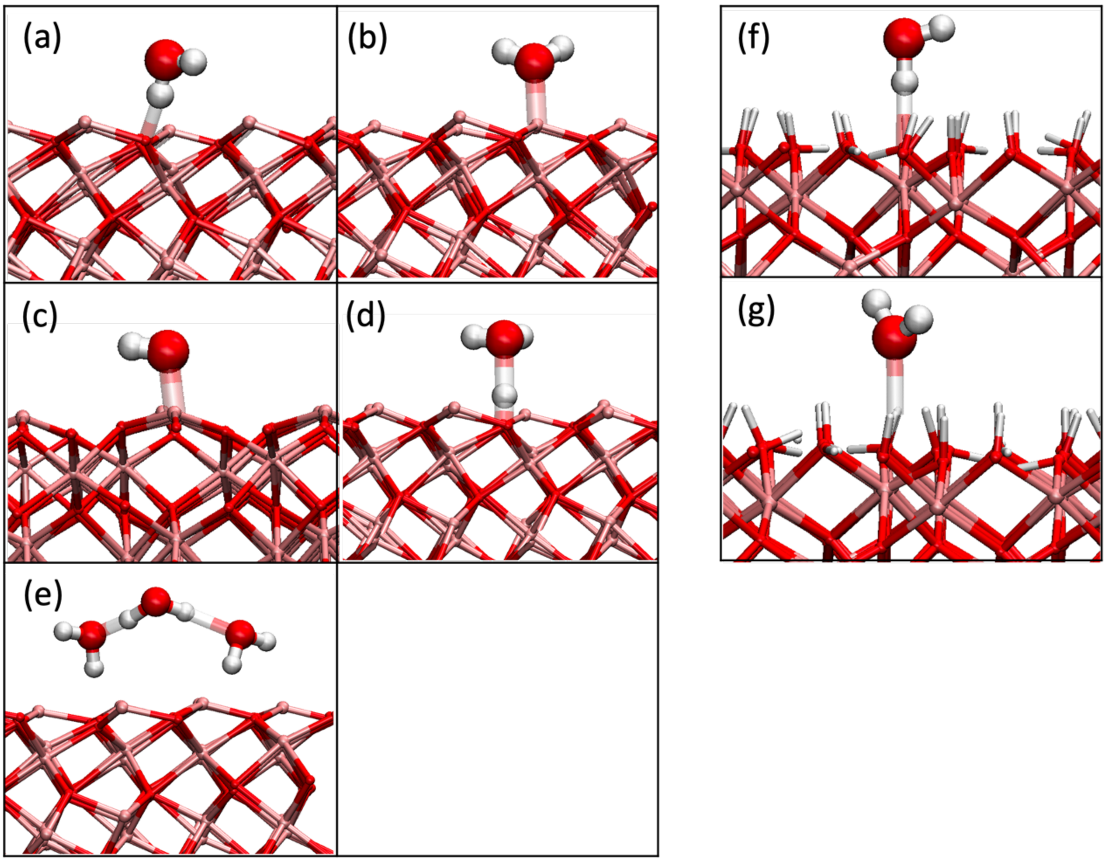

Figure S3. Species in L1 at Al-terminated alumina-water interface (left): (a) water molecules adsorbed on surface O atoms, (b)water molecules adsorbed on the surface Al atoms, (v) adsorbate hydroxide ions, (d)water adsorbed on surface aluminol, and (e)water molecules forming hydrogen bonds with the adsorbed water. Species in L1 at O-terminated alumina-water interface (right): (f) water molecules adsorbed on O atom in surface aluminol, (g) water molecules adsorbed on the H atom in surface aluminol. O atoms in red, H atoms in white and Al atoms in pink.

Water species in L1 layer at the Al-terminated and O-terminated alumina-water interfaces are taken from the AIMD trajectories and shown in Figure S3. L1 in Al-terminated system is composed of all five water species(a-e), while f is the dominant species in the O-terminated system.

4. Definition of Tetrahedral Order Parameter

The orientational tetrahedral order parameter Q, which is one of the most widely used tetrahedral order parameters to characterize the tetrahedral arrangement of the local structure of liquid water, is defined as:[2]

$$Q = 1 - \frac{3}{8}\sum_{j=1}^{3}\sum_{k=j+1}^{4}(cos\Psi_{j,O,k} + \frac{1}{3})^2 ,$$

where $\Psi_{j,O,k}$ is the angle between the two lines joining the oxygen atom of the water under consideration and two of its four nearest (heavy atom) neighbors (see in Figure S1). Here the heavy atoms include the O atom from the water, and O and Al in the alumina slab. For a regular tetrahedron the value is 1.

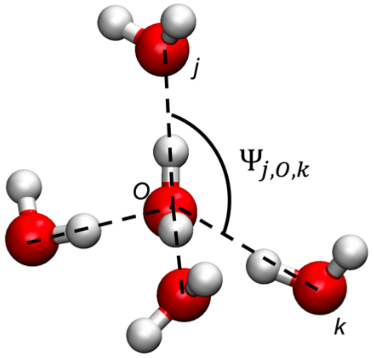

Figure S4. Illustration of angle $\Psi_{j,O,k}$ for the calculation of orientational tetrahedral order parameter Q. Red and white balls represent water O atoms and H atoms respectively. O is the water O under consideration; j and k are the two of its four nearest heavy atoms.

5. **Simulated vSFG spectra of the surface aluminols at the O-terminated alumina-water interface**

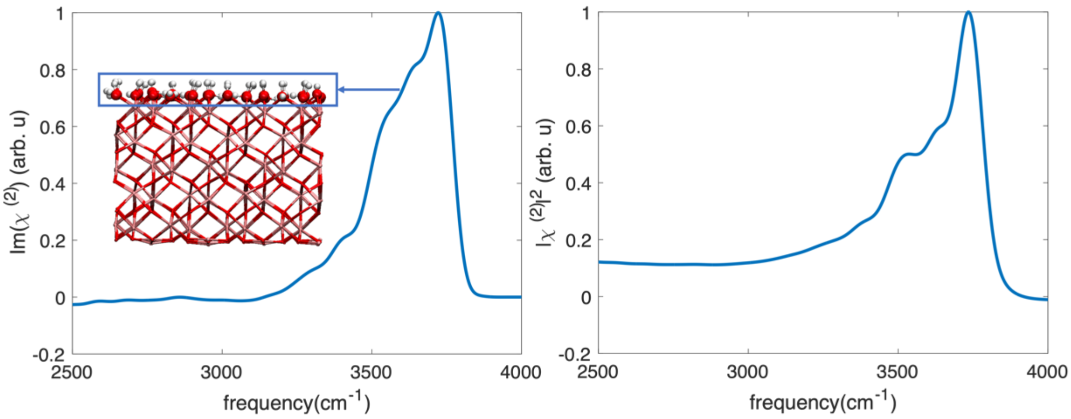

Figure S5. Simulated vSFG spectra(left) of aluminols at O-terminated water-alumina interfaces and its imaginary part (right). An illustration of the O-terminated alumina slab is inserted in the left panel. The O, H, and Al atoms are represented in red, white, and pink respectively.

Surface aluminols in the O-terminated alumina slab are shown using a ball-and-stick model in the blue box in the inserted panel. The simulated vSFG spectra and the imaginary part of the spectra using only those surface aluminols show sharp positive peak centered at 3700 cm^{-1} with a subtle shoulder at around 3500 cm^{-1}. The high frequency peak (3700 cm^{-1}) arises from the aluminol perpendicular to the alumina-water interface, while the shoulder (3500 cm^{-1}) is generated by the parallel aluminols. The z component of the transition dipole moment of the parallel aluminols is close to 0, so the intensity of the 3500 cm^{-1} peak is low.

6. Frequency calculations of the 2D and 3D water at the Al-terminated alumina-water interface

Two representative clusters were taken from AIMD simulations that had the dominant hydrogen bonding environment for a 2-up water at the Al-terminated interface, namely 2_D and 3_D, respectively. The clusters with the two OH stretching mode, OH1 and OH2, are shown in the blue circle in the water clusters shown in Figure S3. The potential energy surface of the OH stretch mode is obtained by scanning the hydrogen along the direction of the investigated OH bond. The calculations are performed using Gaussian 16 software at DFT level using the B3LYP functional and 6-31+g(d,p) basis set. The potential of the energy surface (shown in Figure S4) is then fit to the to the Morse potential energy function[3], and the frequencies are calculated and tabulated in Table S1.

From Figure S4, it is observed that the proton in 2_D: OH1 and 3_D: OH2 are relatively delocalized. The simulated frequencies of these two bonds are 2633 cm^{-1} and 3043 cm^{-1}, in agreement with simulated vSFG spectra of OH stretching for 2-up water in layer L1 at the Al-terminated alumina-water interface. The localized hydrogens in in 2_D: OH2 and 3_D: OH1 show frequencies at 3260 cm^{-1} and 3180 cm^{-1} respectively, which is also observed in the simulated vSFG spectra.

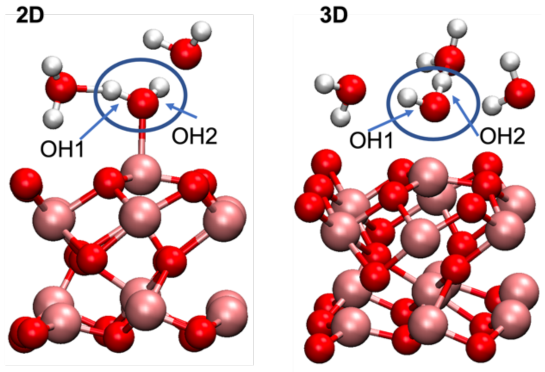

Figure S6. Representative Al-terminated water-alumina cluster taken from the AIMD trajectory showing the 2-up 2D (left) and 3D (right) water.

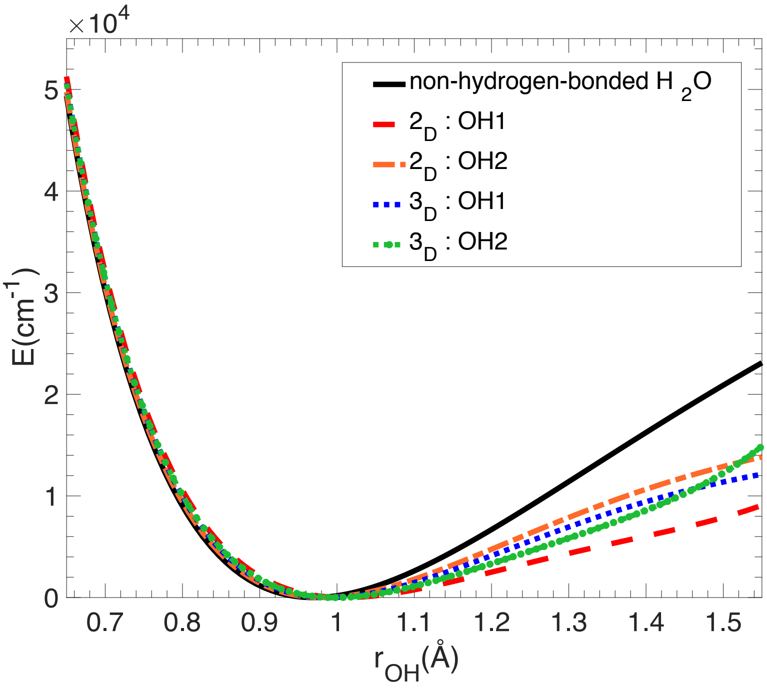

Figure S7. Potential of energy surface of OH stretching mode for 2D and 3D water at the Al-terminated alumina-water interface.

Table S1. The calculated frequencies for the OH stretching modes shown in Figure S3.

OH stretching mode	Frequency(cm^{-1})
2_D: OH1	2633
2_D: OH2	3260
3_D: OH1	3043
3_D: OH2	3180

7. References

1. Nygren, M.A.; Gay, H. D.; Richard, C.; Catlow, A., Hydroxylation of the surface of the corundum basal plane. *Surface Science* **1997**, 380 (1), 113-123.
2. Duboué-Dijon, E.; Laage, D., Characterization of the Local Structure in Liquid Water by Various Order Parameters. *The Journal of Physical Chemistry B* **2015**, 119 (26), 8406-8418.
3. Corcelli, S.; Lawrence, C.; Skinner, J. L., Combined electronic structure/molecular dynamics approach for ultrafast infrared spectroscopy of dilute HOD in liquid H 2 O and D 2 O. *Journal of Chemical Physics* **2004**, 120 (17), 8107-8117.
